# Application of artificial intelligence in X-ray imaging analysis for knee arthroplasty: A systematic review

**DOI:** 10.1371/journal.pone.0321104

**Published:** 2025-05-07

**Authors:** Zhihong Zhang, Xu Hui, Huimin Tao, Zhenjiang Fu, Zaili Cai, Sheng Zhou, Kehu Yang

**Affiliations:** 1 Department of The First Clinical Medical College of Gansu, University of Chinese Medicine, Lanzhou, Gansu, China; 2 Department of Evidence-Based Medicine Centre, School of Basic Medical Science, Lanzhou University, Lanzhou, Gansu, China; 3 Department of Centre for Evidence-Based Social Science/Center for Health Technology Assessment, School of Public Health, Lanzhou University, Lanzhou, Gansu, China; 4 Department of Gansu Key Laboratory of Evidence-Based Medicine, Lanzhou University, Lanzhou, Gansu, China; 5 Department of Radiology, Renhuai People’s Hospital, Zuiyi, Guizhou, China; 6 Department of Radiology, Gansu Provincial Hospital, Lanzhou, Gansu, China; Southern Medical University Nanfang Hospital, CHINA

## Abstract

**Background:**

Artificial intelligence (AI) is a promising and powerful technology with increasing use in orthopedics. The global morbidity of knee arthroplasty is expanding. This study investigated the use of AI algorithms to review radiographs of knee arthroplasty.

**Methods:**

The Ovid-Embase, Web of Science, Cochrane Library, PubMed, China National Knowledge Infrastructure (CNKI), WeiPu (VIP), WanFang, and China Biology Medicine (CBM) databases were systematically screened from inception to March 2024 (PROSPERO study protocol registration: CRD42024507549). The quality assessment of the diagnostic accuracy studies tool assessed the risk of bias.

**Results:**

A total of 21 studies were included in the analysis. Of these, 10 studies identified and classified implant brands, 6 measured implant size and component alignment, 3 detected implant loosening, and 2 diagnosed prosthetic joint infections (PJI). For classifying and identifying implant brands, 5 studies demonstrated near-perfect prediction with an area under the curve (AUC) ranging from 0.98 to 1.0, and 10 achieved accuracy (ACC) between 96–100%. Regarding implant measurement, one study showed an AUC of 0.62, and two others exhibited over 80% ACC in determining component sizes. Moreover, Artificial intelligence showed good to excellent reliability across all angles in three separate studies (Intraclass Correlation Coefficient > 0.78). In predicting PJI, one study achieved an AUC of 0.91 with a corresponding ACC of 90.5%, while another reported a positive predictive value ranging from 75% to 85%. For detecting implant loosening, the AUC was found to be at least as high as 0.976 with ACC ranging from 85.8% to 97.5%.

**Conclusions:**

These studies show that AI is promising in recognizing implants in knee arthroplasty. Future research should follow a rigorous approach to AI development, with comprehensive and transparent reporting of methods and the creation of open-source software programs and commercial tools that can provide clinicians with objective clinical decisions.

## Introduction

Knee arthroplasty surgeries were initially widely performed in the 1,970s and 1,980s and are now considered an effective and cost-efficient treatment for end-stage knee arthritis [1]. It is projected that total knee arthroplasty (TKA) alone will exceed 1.26 million annual cases in the United States [2]. As the population of arthroplasty patients continues to grow, healthcare systems will face an increasing burden [3]. Therefore, understanding the causes and risk factors of revision knee arthroplasty to enhance surgery longevity has become increasingly important. Consequently, conducting postoperative imaging examinations to evaluate prosthesis characteristics and detect abnormalities is crucial. Various imaging techniques like X-ray, MRI, arthrography, scintigraphy, and fluorodeoxyglucose-positron emission tomography (FDG-PET) have been extensively used [4,5]. Among these methods, X-ray examination is considered primary due to advantages such as shorter duration of examination, cost-effectiveness, and enhanced diagnostic accuracy [6,7].

Due to recent developments, AI diagnostic capabilities have gained significant attention as an efficient and standardized tool in medicine and orthopedics [8]. Machine learning (ML) and deep learning (DL) models, known as convolutional neural networks (CNNs), are subsets of AI modeled after the human brain to identify rules and patterns in images [8–10]. AI algorithms have been reported to automatically diagnose stroke, retinopathy, and cancer histology with the same accuracy as experts in related fields [11–14]. Due to its clear operational criteria and internal logic involved in knee prosthesis selection, AI techniques can effectively model this process. In recent years, there has been a significant increase in AI research for analyzing various aspects of knee arthroplasty, including implant identification, implant failure, precise measurement of dimensions, hospitalization duration, potential complications, cost-effectiveness analysis, functional recovery outcomes, and optimal surgical techniques [15–20]. However, many of these algorithms were either underpowered or included misleading parameters. Importantly, medical imaging plays a crucial role in providing clinicians with convenient access to patient information in real healthcare settings. By utilizing labeled radiographs from medical experts, AI has emerged as a powerful and effective method for musculoskeletal imaging [21].

Consequently, we conducted a comprehensive systematic review of all relevant studies. This study focused on the application of X-ray imaging-based AI in prosthesis-related studies of knee arthroplasty and explored the diagnostic performance and analysis of AI algorithms and models for knee prostheses.

## Materials and methods

This study was conducted following the guidelines in the Preferred Reporting Items for Systematic Reviews and Meta-Analyses of Diagnostic Test Accuracy (PRISMA-DTA) statement [22], and was prospectively registered on the International Prospective Register of Systematic Reviews (PROSPERO, CRD42024507549) [23].

### Search strategy

From their inception until July 2023, a comprehensive search was conducted across the electronic databases of CNKI, VIP, Wan Fang, CBM, PubMed, Cochrane Library, Ovid-Embase, and Web of Science, with subsequent updates conducted until March 2024. The keywords used in the search strategy included (“knee arthroplasty” OR “knee prosthesis” OR “knee replacement”) (“prosthesis” OR “implants”) and (“artificial intelligence” OR “deep learning” OR “machine intelligence” OR “machine learning” OR “computational intelligence” OR “AI” OR “algorithms” OR “random forest” OR “support vector machine” OR “decision trees” OR “artificial neural network” OR “radiomics”). Furthermore, we manually screened the reference lists of the included studies to identify any publications that may be included in our systematic search. No limitations were imposed on the studies’ source or the publications’ language. The detailed search strategy is provided in S1 Table.

### Study selection

Studies were imported into EndNote 20 and Rayyan (https://www.rayyan.ai/) for screening. Title and abstract screening, and full-text screening were performed independently by two reviewers, and discrepancies were resolved through discussion. Studies included met these criteria: 1) Study types: cohort studies and cross-sectional studies; 2) Participants: with or without knee prosthesis; 3) Index tests: diagnosis of knee arthroplasty based on quantitative imaging data (X-rays) using AI algorithms; 4) Reference standards: patients with a diagnosis confirmed by medical guidelines, clinical surgical records; 5) Outcomes: ultimately, we identified the currently established use four cases for AI in knee arthroplasty implant analysis. We excluded inaccessible studies, conference abstracts, reviews, editorials, consensus, guidelines, meta-analyses, duplicated studies, or review articles.

### Data extraction

Two reviewers independently performed data extraction (ZZH and THM), from August to September 2023, and disagreements were resolved through discussion with a third reviewer(HX). When data were insufficient or missing from the articles, we attempted to contact the study authors for additional information. The following variables were included: 1) study characteristics: first author, publication year, country, prediction type; 2) patient clinical characteristics: number of samples, demographics; 3) model characteristics: index tests, reference standards, data sources, radiographs samples, implant designs, the proportion of data sets, external verification, AI device, AI method, AI techniques; 4) diagnostic performance measures such as the area under the curve (AUC), accuracy (ACC), sensitivity (SEN), specificity (SPE), positive predictive value (PPV), negative predictive value (NPV), or intraclass correlation coefficient (ICC).

### Bias assessment and certainty of evidence

The Quality Assessment of Diagnostic Accuracy Studies-AI (QUADAS-AI) tool is an expanded instrument that incorporates the characteristics of AI research based on the QUADAS-2 [24] and QUADAS-C tools [25], which supplement the bias risk assessment in four relevant domains: case selection, diagnostic tests, reference standards, research processes, and timing. However, it should be noted that the current version of the QUADAS-AI tool is still in draft form and has not yet reached maturity regarding signal problems. Therefore, the QUADAS-2 tool was chosen as the preferred method for assessing bias risk. The tool includes four domains for risk of bias assessment and three for applicability concerns. Each section is classified as having a high, low, or unclear risk of bias. Two reviewers independently conducted a risk of bias assessment using the QUADAS-2 tool [24], and a third reviewer consulted to achieve consensus on each item. Furthermore, Review Manager 5.3 software aided in visually portraying the risk of bias assessment, providing a concise and intuitive representation of the results [26].

### Data analyses

Descriptive statistics were employed to succinctly summarize the noteworthy findings and outcomes of the selected studies, as well as to delineate prevailing trends in AI technology, clinical applications, and their associated discoveries. Summary data were presented employing elementary means, frequencies, and proportions. The performance of AI models in the assessed studies was summarized utilizing diverse metrics encompassing AUC, ACC, SEN, SPE, PPV, NPV, and ICC.

## Result

### Search results

**Fig 1:**
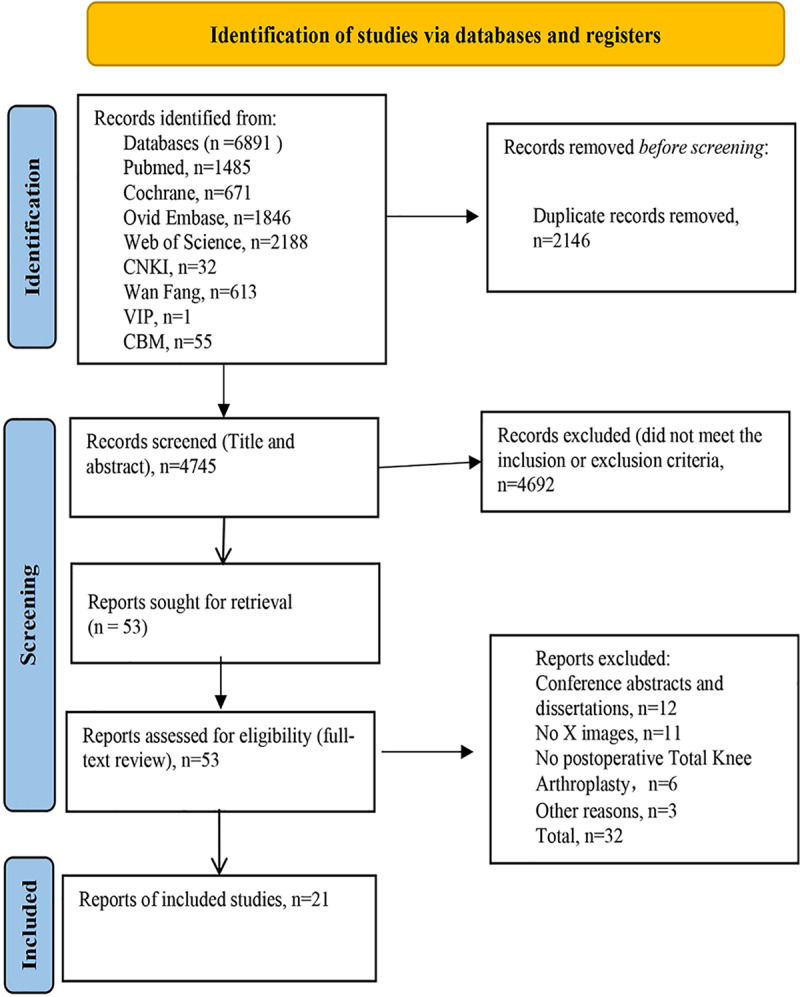
PRISMA flow chart of the literature retrieval.

A study-flow diagram shows the process for study identification, inclusion, and exclusion (Fig 1). A comprehensive search yielded 7083 studies, which were subsequently screened for duplicates resulting in 4937 unique studies. After evaluating titles and abstracts, we excluded 4884 studies that did not meet the predefined inclusion/exclusion criteria. Fifty-three full-text articles underwent eligibility assessment, leading to the exclusion of 32 studies. Ultimately, a total of 21 studies met the criteria and were included in this systematic review.

### Characteristics of thṭe included studies

Of the 21 studies evaluating range from tens to over ten thousand radiographs were included, with 10 studies explored AI application in classification of manufacturers and brands [27–36], and 6 studies focused on implant measurements, including implant size and component position, alignment [37–42], 3 studies were concerned with the detection of implant loosening [43–45], and 2 studies related to the prediction and diagnosis of prosthetic joint infections (PJI) [46,47]. All studies were published during 2020–2023. All studies were retrospective studies. There were 6 studies involving various AI models [30,31,33,35,43,47], and others used a single AI model. There were 13 image-related studies used CNN models [27–32,34], 4 used ML models [33,37,38,45], 3 used AI tools [36,40,42], and 1 used algorithms [46]. Also 7 studies used widely learned CNN models from Imagenet [27,28,31,43–45,47]. 2 studies utilized ResNet [27,28], 4 studies employed Inception V3 [29,32,34,44]. 4 studies analyzed various AI models and selected those with the highest accuracy, namely DenseNet [31,43], Efficient Net & U-Net [30], MobileNetV2 [35], and the VGG-16 transfer learning Model [33]. Additionally, focusing on the model’s architecture methods like dropout regularization [45], batch normalization [28–30,32,34,45], transfer learning [27,28,31,33,41,43,45], pretraining [27,43], and access to a large number of high-quality image datasets [27,28,31,43–45,47] in this study. Thirteen studies data originated from a single center [27,30,32,36–39,41–44,47,48], while 8 studies drew from multiple centers [28,29,31,33–35,40,46]. Seven studies were based on US populations [27,29,32,34,37,43,46]; 3 studies originated from China [41,44,47], India [31,33,35], and the UK [28,30,38], respectively; 2 studies from Germany [39,42]; and one from France [36], Australia [40], and Korea [45], respectively. The number of implants brand each algorithm could classify varied between the studies, with certain studies failing to disclose the number of prostheses [39,42–47], while the highest reported count was 14 prosthesis manufacturers [32]. We also observed that the most commonly used X-ray imaging positions among the included studies were anteroposterior (AP) and lateral (LAT) views (86%). The detailed features of the studies are summarized in Table 1.

**Table 1:** Characteristics of the included studies.

Author	Index test	Number of Samples	Data Sources (center)	Reference Standards	Demographics			Train/ validation/ Test	External test set
					Female (%)	Avg. age	Avg. BMI		
Implant Identification and Classification									
Yi 2020 [27]	AP & LAT Radiograph	511^a^	Single	Clinician evaluations	NR	NR	NR	7:1:2	No
Ghose 2020 [35]	AP & LAT Radiograph	878^a^	Multiple	Implant manufacturers	NR	NR	NR	8:1:1	No
Belete 2021 [28]	AP Radiograph	588^a^	Multiple	Trained surgeons	NR	NR	NR	5:2.5:2.5	No
Karnuta 2021 [29]	AP Radiograph	682^a^	Multiple	Medical records	NR	NR	NR	8:1:1	Yes
Patel 2021 [30]	AP Radiograph	427^a^	Single	Surgical records	NR	NR	NR	7:2:1	No
Sharma 2021 [31]	AP & LAT Radiograph	1078^a^	Multiple	Orthopedic surgeons	NR	NR	NR	7.5:1.5:1	Yes
Klemt 2022 [32]	AP Radiograph	4722^a^	Single	Medical records	NR	NR	NR	8:2:0	No
Tiwari 2022 [33]	AP & LAT Radiograph	521^a^	Multiple	Surgical records	NR	NR	NR	7:2:1	No
Karnuta 2023 [34]	AP Radiograph	4724^a^	Multiple	Medical records	NR	NR	NR	8:1:1	Yes
Bonnin 2023 [36]	AP & LAT Radiograph	39751^a^	Single	Implant manufacturers	48	62	NR	6:2:2	No
Implant Measurement									
Farooq 2021 [37]	Weight-bearing, LAT Radiograph	1091^a^	Single	Clinical scoring guidelines	67	66	34	NR	No
Erne 2022 [39]	Weight-bearing, LAT Radiograph	200^a^	Single	Clinicians evaluations	39	66	NR	NR	Yes
Schwarz1 2022 [40]	LLR	1651^a^	Multiple	Clinicians evaluations	67	69	NR	75:1:7	No
Pagano 2023 [42]	AP Radiograph	200^a^	Single	Clinical referencebenchmarks	50	67	30	NR	No
Yue 2022 [41]	AP & LAT Radiograph & Demographic data	308^a^	Single	Actual sizing OR Surgical records	78	NR	NR	4:1:1	No
Burge 2022 [38]	AP & LAT Radiograph & MRI	176/78^a^	Single	Actual sizing ORSurgical records	58	62	NR	9:1:4	No
Prosthetic Joint Infection									
Weinstein 2021 [46]	X-ray; Clinical information	80^b^	Multiple	Medical records	NR	NR	NR	NR	No
Wu 2022 [47]	AP & LAT Radiograph	1062/531^b^	Single	The MSIS Criteria	60	64	NR	8:2:0	No
Implant Loosening									
Shah 2020 [43]	AP & LAT Radiograph & Demographic & comorbidity data	217^b^ (Fixed) 137^b^ (Loose)	Single	Operations records	54(Fixed) 56(Loose)	67(Fixed) 69(Loose)	NR	6:2:2	No
Lau 2022 [44]	X-ray & Clinical information	234^b^ (Fixed) 206^b^ (Loose)	Single	Operations records	NR	NR	NR	NR: 1:3	No
Kim 2023 [45]	AP Radiograph	100^b^ (Fixed) 100^b^ (Loose)	Single	Imaging & Operation records	80(Fixed) 80(Loose)	71(Fixed) 70(Loose)	27(Fixed) 26(Loose)	8:NR:2	No

AI: artificial intelligence; DCNN: Deep Convolutional Neural Network; NR, not reported; AP radiographs: Anterior-Posterior Radiographs; LAT radiographs: Lateral Radiographs; BMI: Body Mass Index; MRI: Magnetic Resonance Imaging; ML: Machine learning; aNumber of radiograph images; bNumber of participants; LLR: Long Leg Radiog

### Identification and classification of implants

These included 10 studies [27–36] (Table 2). The first study by Yi et al [27] in 2020 successfully demonstrated CNNs’ ability to accurately identify the presence of TKA, distinguishing between TKA and UKA, and two different primary TKA models on knee radiographs. All achieved an AUC of 1 and a SEN of 100%. In 2021, Belete et al [28] proposed a comparable CNN that effectively distinguished between TKA implants and no prosthesis, achieving an impressive ACC of 100% and an AUC score of 100%. Additionally, they developed a deep CNN for automatic classification of 7 types of knee arthroplasties on radiographs, demonstrating a remarkable ACC rate of 100%. Patel et al [30] conducted a study where they developed classification networks to identify 12 designs of hip and knee implants from radiographs. The final network achieved an impressive accuracy rate of 98.9%, outperforming all five specialists (median ACC rate of 76.1% and best ACC rate of 85.6%). Another study by Klemt et al [32] demonstrated that their AI model successfully distinguished between 24 THA and 14 TKA designs and became the first to identify primary and revision TKA designs automatically. Furthermore, this study had the largest sample size among all types of TKA implants examined. Their CNN model exhibited excellent discrimination in identifying eight primary TKA designs with an AUC value of 0.97 and six revision TKA designs with an AUC value of 0.96. Sharma et al [31] utilized a DL network trained on 1078 radiographs to accurately distinguish among six knee arthroplasty implants, with an internally validated AUC value of 0.98. Moreover, two studies [35,36] achieved similar precision in identifying automated knee implants, with ACCs reaching 96.7% and 99.9%, respectively. Through internal validation on plain radiographs, Tiwari et al [35] reported the development of an algorithm capable of identifying seven distinct knee arthroplasty models with an ACC rate of 95%. The study further highlighted that ML outperformed two human experts who achieved only an average ACC of 78%. The internally validated study by Karnuta et al [29] in 2021 effectively discriminated nine different knee arthroplasty implant models using 682 radiographs, yielding near-perfect AUC values ranging from UKAs to TKAs to distal femoral replacements (DFRs). Karnuta et al [36] conducted external validation in 2023 to enhance clinical applicability and ensure generalizability, significantly expanding the dataset to include 4724 radiographs classified based on a previous study [29]. The externally validated model exhibited outstanding performance with an AUC of 0.989 while maintaining an impressive average processing speed of 0.02 seconds per image for implant identification. Karnuta’s study [36] also identified nine knee prosthesis system designs from four manufacturers, representing contemporary and commonly used models.

**Table 2:** Performance of AI algorithms iin identifying implant in knee arthroplasty.

Author	Identifying objects	Implant designs	AI Method	AI Techniques	Diagnostic performance					
					AUC	ACC (%)	SEN (%)	SPC (%)	PPV (%)	NPV (%)
Yi 2020 [27]	Presence or Absence of TKA; TKAs & UKA	3	ResNet-18 ResNet-152	The Solver Parameters, Data Augmentation, Heatmaps	1.00 1.00	100.00 100.00	100.00 100.00	100.00 100.00	100.00 100.00	100.00 100.00
Ghose 2020 [35]	TKAs	6	MobileNetV2 (Best model)	Histogram Equalization, Data Augmentation, Albumentations, Class Activation Map	NR	96.70	NR	NR	NR	NR
Belete 2021 [28]	Absence of prosthesis & TKAs	7	ResNet-18	Hyperparameter, Manual Segmentation Pre-Processing, Data Augmentation, Saliency mapping	1.00	100.00	100.00	NR	NR	NR
Karnuta2021 [29]	TKAs & UKA	9	Inception V3	Image Preprocessing, CNN Models, Class Activation Heatmap	0.99	99.00	95.00	99.00	90.00	99.00
Patel 2021 [30]	HKAs & TKAs & UKA	4	Efficient Net & U-Net (Best model)	Classification Network Architectures, Hyperparameter Optimization, Image Segmentation, Ensembled Network, Saliency Mapping, Data Augmentation	NR	98.90	98.90	NR	99.00	NR
Sharma 2021 [31]	TKA	6	DenseNet201 (Best model)	BRISQUE, Data Augmentation, Fine-Tuning in Transfer Learning, Saliency Map	0.99	96.38	97.20	NR	NR	NR
Klemt 2022 [32]	Primary THAs & TKAs, Revision THAs & TKAs	14	Inception V3	Image Preprocessing, Hyperparameter Optimization, Class Activation Heat Maps	NR	97.40^a^ 96.30^b^	94.90^a^ 94.50^b^	97.80^a^ 98.10^b^	NR	NR
Tiwari 2022 [33]	TKA	6	VGG-16 transfer learning Model (Best model)	Transfer Machine Learning Models, Data Augmentation,	NR	95.50	93.50	98.40	NR	NR
Karnuta 2023 [34]	UKA & TKAs & DFR	9	Inception V3	Image Preprocessing, Hyperparameter Optimization, Class Activation Heatmap, Data Augmentation	0.99	97.40	89.20	99.00	NR	NR
Bonnin 2023 [36]	TKA	4	X-TKA	Exam Quality Control CNN (12 DCNN Algorithms)	NR	99.90	99.80	100.00	100.00	NR

NR: not reported; BRISQUE: Blind Reference less Image Spatial Quality Evaluator.; AUC: the area under the curve; ACC: accuracy; SEN: sensitivity; SPE: specificity; PPV: positive predictive value; NPV: negative predictive value; UKA: unicompartmental knee arthroplasties; TKA: Total Knee Arthroplasty; DFR: distal femoral replacements. aPrimary TKA; bRevision TKA.

### Implant measurement

These included 6 AI studies to assess knee prosthesis angle alignment, position, and size (Table 3).

**Table 3:** Performance of AI algorithms in measuring implant in knee arthroplasty.

Author	AI Models	AI Techniques	Diagnostic Performance	
			AUC/ICC/ACC (%)	Other outcomes
Farooq 2021 [37]	ML	A Tree Net Gradient Boosting Machine	AUC: 0.62	“Satisfied or very satisfied” and a knee “always feeling normal”: closer to native -2° to +2(tibial slope change), 0–7° (femoral component flexion)); Worse outcomes: changing>5° (native tibial slope), beyond +10(any femoral component extension).
Erne 2022 [39]	AI-based Algorithm	Preprocessing; Landmark Placement; Landmark Placement; Projection and Parameter Computation	ICC: Human vs. human: 0.85–0.99(inter-rater);0.95–1.0(intra-rater); AI vs mean human: 0.80–1.00(Preoperative);0.83–0.99(Postoperative).	The detection rate for the different angles ranged between 92.40% and 98.90%.
Schwarz 2022 [40]	IB Lab LAMA	LAMA; Leg Angle Measurement Assistant, version 1.03(IB Lab GmbH,Vienna, Austria)	ICC: 0.99(HKA); 0.99(FCA) 0.97(TCA).	The AI software was reproducible on 96.00% and reliable on 92.10% of LLRs; 0.2°varus ± 2.5° (Postoperative HKA); 89.3° ± 1.9° (Postoperative FCA); 89.1° ± 1.6° (Postoperative TCA); 1.6 varus ± 6.4° (Preoperative HKA); 90.5° ± 3.1° (Preoperative FCA); 88.9° ± 4.1° (Preoperative TCA).
Pagano 2023 [42]	IB Lab LAMA	LAMA, Version1.13.16 (September 2022, IB Lab GmbH, Vienna, Austria)	ICC: 1.00(MAD), 0.93(mLPFA), 0.87(mLDFA), 0.86(mMPTA), 0.95(mLDTA),0.99(HKA), 0.78(Mikulicz line), 0.79(JLCA);0.81(AMA)	NR
Yu 2022 [41]	ECOC-based Models	Baseline Predictive Modeling (ResNet18); ECOC-Based Optimization	ACC: 88.23(femoral component); 86.27(tibial component).	NR

AI: artificial intelligence; DCNN: Deep Convolutional Neural Network; NR, not reported; AP radiographs: Anterior-Posterior Radiographs; LAT radiographs: Lateral Radiographs; BMI: Body Mass Index; MRI: Magnetic Resonance Imaging; ML: Machine learning; *: Number of participants; #: Number of radiograph images; LLR: Long Leg Radiograph; NR: not reported.

### Implant measurement-alignment

Among 4 studies employed AI to evaluate component alignment accuracy in TKA [37,39,40,42]. Farooq et al [37] used ML to predict clinical knee improvement based on sagittal implant location. ML models showed high satisfaction and normal knee function within -2° to +2° tibial slope and 0° to +7° femoral component flexion. Conversely, worse outcomes were predicted with femoral component extension, excessive flexion (>+10°), or significant alteration (>5°) of the native tibial slope. Erne et al [39] developed an innovative AI algorithm for determining lower limb alignment angles with a detection rate ranging from 92.4% to 98.9%. The excellent reliability and agreement between human vs human comparisons as well as AI vs mean human comparisons (ICC range: 0.8–1.0) highlight the outstanding performance of AI algorithms in terms of assessment speed (<60 seconds) and ACC for evaluating lower extremity alignment. Two studies [40,42] demonstrated the effectiveness and reliability of AI-powered applications (IB Lab LAMA) for automated measurement of lower limb alignment, which outperformed traditional orthopedic measurements by providing results in just 20 seconds per radiograph analysis using AI software alone. Schwarz et al.’ study [40] revealed a strong correlation between manual reads and AI software measurements for hip-knee-ankle (HKA), femoral component angle (FCA), and tibial component angle (TCA) (ICC>0.97). Additionally, the repeatability (96%) and reliability (78%) of long-leg radiographs with TKA assessed by the AI software further supported its efficacy. Pagano’s study [42], complemented by studies from Schwarz et al. [42], assessed more parameters using AI-powered software, including axial alignments, femoral and tibial angles, as well as other vital measurements such as joint-line convergence angle (JLCA), HKA, and Mikulicz line

### Implant measurement-dimensions

With 2 studies evaluated the implant dimensions using AI [38,41]. Yue et al [41] employed an ECOC-based CNN model that incorporated patient gender and BMI with X-ray images to achieve prediction ACC of 88.23% and 86.27% for femoral and tibial components, respectively. Burge et al [38] evaluated the performance utility of an ML-based 2D-3D pipeline, which accurately predicted distal femur and proximal tibia sizes from X-ray images. The authors demonstrated that their model achieved an average root mean square error (RMSE) percentage of 77.9% and 80.5% for femoral and tibial component sizes while achieving accuracy rates of 71.8% for both maximum up/down hanging measurements, representing improvements by approximately 99.5% and 99.9%, respectively, within the ±1 size metric.

### PJI of knee arthroplasty

Two studies predicted PJI using X-rays based on AI algorithms [46,47] (Table 4). One study [46] developed diagnostic coding algorithms using the International Classification of Diseases, 9th Revision (ICD-9), International Classification of Diseases, 10th Revision (ICD-10), and current procedural terminology (CPT) codes to identify PJI for knee arthroplasty in the US Veterans Health Administration. The study achieved PPVs of 75% (ICD-9) and 85% (ICD-10) for confirmed PJI events, which could be helpful for future pharmacoepidemiologic studies. Another study [47] developed DL models for classification networks using 1062 dual-channel X-ray images, which can assist clinicians in identifying PJI and aseptic failure with high SEN and SPE. It achieved an AUC of 0.931, a SEN of 0.905, and a SPE of 0.889 for identifying PJI in knee prostheses. Moreover, the high-risk regions predicted by the PJI deep learning model were closely correlated with intraoperative clinical and pathological findings

**Table 4:** Performance of AI Algorithms in predicting joint infection in knee arthroplasty.

Author	AI Models	AI Techniques	Diagnostic Performance				
			AUC	ACC (%)	SPE (%)	PPV (%)	NPV (%)
Weinstein 2021 [46]	ICD-9-based and ICD-10-based case-finding algorithms	Algorithms consisted of ICD-9 or ICD-10 PJI diagnosis & TKA code & CPT codes for a knee X-ray & relevant surgical procedure & microbiologic culture	NR	NR	NR	75.00(ICD-9 PJI) 85.00(ICD-10 PJI)	NR
Wu 2022 [47]	DCNN	Classification Network (Consisted of VGG16. Inception-v3. Resnet-50. DenseNet-121); Best Hyperparameter Interpret Network (heatmaps)	0.91	90.50	88.90	93.10	84.60

NR: not reported; CPT: current procedural terminology; ICD-9: International Classification of Diseases, 9th revision; ICD-10: International Classification of Diseases, 10th Revision; PJI: Prosthetic joint infection; DCNN: deep convolutional neural networks; AUC: the area under the curve; ACC: accuracy; SPE: specificity; PPV: positive predictive value; NPV: negative predictive value.

### Implant loosening

Included 3 studies have monitored the implant loosening in TKA [43–45] (Tabel 5). Shah et al. [45] evaluated a model for detecting loosening using 217 fixed TKA and 137 loosened TKA X-rays, employing various CNN models. The transfer learning approach utilized image modification techniques without altering the layers. The ACC achieved 70.8% when using original images with untrained models, which improved to 73.3% when segmentation and cropping tools were applied and exceeded 80% when pre-trained models with large datasets were used. When only images were considered, the ACC remained below 90%; however, incorporating clinical information increased the ACC beyond 90%. Lau et al. [44] conducted a study on TKA loosening analysis involving a dataset of 440 knee radiograph images utilizing an image-based ML model based on Xception architecture without fine-tuning. They achieved an overall ACC of 96.3%, SEN of 96.1%, SPE of 90.9%, and the AUC of 93.5%. Assessment by senior orthopedic specialists yielded an ACC range between 89.09% to 94.54%. Kim et al [45] employed a transfer learning model using VGG19 as a pre-trained CNN model to assess the accuracy in detecting TKA loosening among 100 patients with loosening and another 100 patients without it; two different degrees of freezing ranges were explored for transfer learning modeling techniques. The solely fine-tuned image models demonstrated impressive results with an ACC of 97.5%, perfect SEN of 100%, and SPE of 97.5%.

### Quality assessment

**Figs 2:**
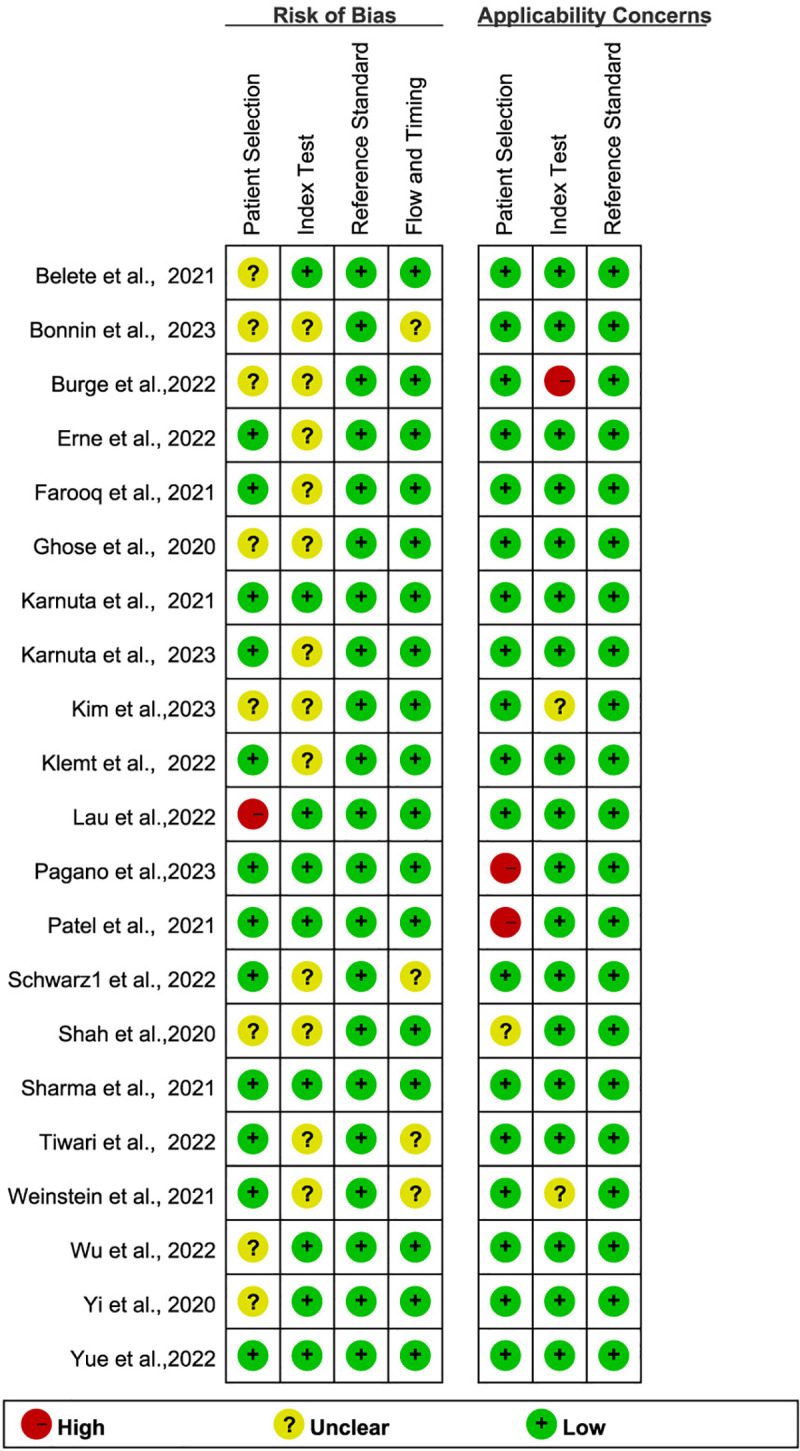
Risk of bias and applicability concerns summary.

**Figs 3:**
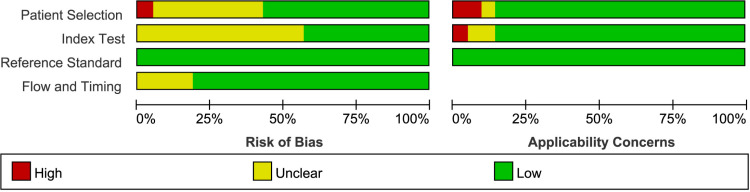
Risk of bias and applicability concerns graph.

The quality assessment of each included study is illustrated in Fig 2-3. In the patient selection domain, nine studies had a high or unclear risk [27,28,35,36,38,43–45,47]. For the index test, 12 studies had an unclear risk [29,32,33,35–40,43,45,46]. No bias was observed in the reference standard domain, while flow and timing biases were high or unclear in 4 studies [33,36,40,46]. Regarding applicability concerns, there were high or unclear applicability issues in the patient selection domain for three studies [30,42,43] and in the index test domain for three studies [38,45,46], and low applicability issues across all studies in the reference standard domain. Most included studies showed a low risk of bias and applicability concerns.

**Table 5:** Performance of AI algorithms in implant loosening in knee arthroplasty.

Author	AI Models	AI Techniques	Diagnostic Performance					
			AUC	ACC (%)	SEN (%)	SPE (%)	PPV (%)	NPV (%)
Shah 2020 [43]	Dense-Net (Best model)	Resize segmentation; Transfer learning	NR	85.80	69.80	95.20	89.50	88.70
Lau 2022 [44]	Xception	Optimization configuration; Class activation maps; Clinical information-based model (Random Forest); Transfer learning	0.94	96.30	96.10	90.90	92.40	95.20
Kim 2023 [45]	Transfer learning model 2 (Best)	Transfer learning models (Fine-tuning); Data augmentation	NR	97.50	100.00	95.00	95.20	100.00

AI: artificial intelligence; NR, not reported; AUC: the area under the curve; ACC: accuracy; SEN: sensitivity; SPE: specificity; PPV: positive predictive value; NPV: negative predictive value

## Discussion

This systematic review aimed to comprehensively assess the research using AI algorithms in various aspects of X-ray-based knee arthroplasty. AI-based models offered a promising solution for identifying knee implant systems from X-radiographs, particularly in scenarios without clinical information. Leveraging object-detection algorithms, these AI algorithms could efficiently analyze images, aiding orthopedic surgeons in resolving complexities and prosthetic challenges associated with knee implants. Thus, they reduced human error and improved treatment workflows [48].

Nonetheless, due to differences in architecture and structure among the AI algorithms used in each study, it is still challenging to objectively and quantitatively compare their diagnostic performance for different types of knee arthroplasty. Therefore, developing and implementing standards for AI research in medicine, such as the machine learning-specific extension of the TRIPOD statement (TRIPOD-ML), is crucial. Despite being recently developed or modified, AI algorithms are expected to have higher accuracy. However, due to the inherent algorithmic differences, no significant variations in accuracy were observed. This suggests that while the AI model itself plays a role, the quantity and quality of the dataset currently hold greater importance [49]. Limited training data samples in some studies employ standard data augmentation methods [27–31,33,34,39,45] and multicenter data sources [28,29,31,33–35,40,46] to improve this situation. The “black box” phenomenon in AI diagnostics is also concerning [50], making models’ internal workings inexplicable. Visualization techniques like class activation mapping [27–32,34,36,44,47,51] are commonly used to highlight essential image areas and provide a visual summary of CNN’s decision-making process. This enhances transparency and ensures immediate understanding for humans. Additionally, the QUADAS-2 tool assessment indicates that most studies in our study have low risks of bias and applicability concerns. However, certain domains like patient selection and the index test exhibit variability, with some studies showing high or unclear dangers and problems. These findings highlight the overall reliability of the included studies while emphasizing areas that may require cautious interpretation due to potential biases or applicability issues.

There are also several potential limitations. Firstly, the methodological heterogeneity of the included studies and variations in the performance reports constrained our survey to a systematic review only. Secondly, the current AI models were trained on a specific population of pre-selected cases, given the retrospective nature of this study. Further validation is imperative to ensure the accuracy and generalizability of these AI models. Thirdly, since all included studies involved predictive AI models, it should be noted that the tools for assessing these models are still under development, particularly with the ongoing extension of the Prediction Model Risk of Bias Assessment Tool for Artificial Intelligence (PROBAST-AI) guidelines. Consequently, the quality evaluation could not be further conducted.

## Conclusion

In conclusion, this study highlights the promising role of AI in analyzing routine X-rays to identify and categorize knee implants, measure dimensions and alignment of knee prostheses, detect PJI, and assess implant loosening. Further research will benefit from including more extensive and diverse datasets, establishing multiple center population databases, and training the algorithms on broader implant designs. Additionally, an essential task for the future of AI is the development of open-source software programs and commercial tools that can provide clinicians with more objective clinical decisions.

## Supporting information

S1 Checklist PRISMA 2020 checklist. (PDF)

S1 Table Search strategy. (PDF)

S1 File Studies identification. (XLSX)

S2 File Evaluation of included studies. (XLSX)
