# Supplementary material for: Application of artificial intelligence in X-ray imaging analysis for knee arthroplasty: A systematic review
Source: PLoS One. 2025 May 7;20(5):e0321104. doi: 10.1371/journal.pone.0321104 (PMC12057988; doi:10.1371/journal.pone.0321104)
Supplement: S1 File — (XLSX) [file pone.0321104.s003.pdf]

Appendix Search Strategy

Table A1. Search Strategy

Database/platforms	Results
CNKI	46
CQVIP	2
WanFang Data	525
CBM	68
PubMed	1465
Cochrane Library	464
Web of Science	2598
EMBASE	1915
TOTAL	7083

Database: CNKI

Retrieval date: March 9, 2024. Results 46

#	Searches（主题：46；篇关摘：34）	Results
#1	(主题:膝关节置换术 + 人工膝关节 + 膝关节成形术 + 膝关节手术 + 膝关节假体 (精确)) AND (主题：假体 + 植入物 (精确)) AND (主题：人工智能 + AI + 深度学习 + 机器学习 + 神经网络 + 影像组学 + 算法 + 辅助算法 + 支持向量机 + 决策树 + 随机森林) (精确))<br>时间范围：发表时间：建库--至今	46

Database: CQVIP

Retrieval date: March 9, 2024. Results 2

#	Searches	Results
#1	(M=膝关节置换术 + 人工膝关节 + 膝关节成形术 + 膝关节手术 + 膝关节假体) and (M=假体 + 植入物) AND (M= (人工智能 + AI + 深度学习 + 机器学习 + 神经网络 + 影像组学 + 算法 + 辅助算法 + 支持向量机 + 决策树 + 随机森林)) AND 年份: 收录起始年--至今	2

Database: WanFang Data

Retrieval date: March 9, 2024. Results 525

#	Searches	Results
#1	(主题:(膝关节置换术 or 人工膝关节 or 膝关节成形术 or 膝关节手术 or 膝关节假体) and 主题:(假体 or 植入物) and 主题:(人工智能 or AI or 深度学习 or 机器学习 or 神经网络 or 影像组学 or 算法 or 计算机辅助诊断 or 支持向量机 or 决策树 or 随机森林))<br>and Date:建库起始-*	525

Database: CBM

Retrieval date: March 9, 2024. Results 68

#	Searches	Results
#1	("关节成形术, 置换, 膝"[不加权:扩展]) OR "人工膝关节"[不加权:扩展]	10666
#2	"膝关节置换术"[常用字段:智能] OR "人工膝关节"[常用字段:智能] OR "膝关节成形术"[常用字段:智能] OR "膝关节手术"[常用字段:智能] OR "膝关节假体"[常用字段:智能]	15033
#3	"假体和植入物"[不加权:扩展]	211405
#4	"假体"[常用字段:智能] OR "植入物"[常用字段:智能]	224540
#5	((((("人工智能"[不加权:扩展])) OR "深度学习"[不加权:扩展]) OR "机器学习"[不加权:扩展]) OR "神经网络, 计算机"[不加权:扩展]) OR "支持向量机"[不加权:扩展]	27020
#6	"人工智能"[常用字段:智能] OR "深度学习"[常用字段:智能] OR "AI"[常用字段:智能] OR "机器学习"[常用字段:智能] OR "神经网络"[常用字段:智能] OR "支持向量机"[常用字段:智能] OR "随机森林"[常用字段:智能] OR "电脑辅助诊断"[常用字段:智能]	46233
#7	(#1) OR (#2)	15894
#8	(#3) OR (#4)	224540
#9	(#5) OR (#6)	46233
#10	(#9) AND (#7) AND (#8)	68

Database: PubMed

Retrieval date: March 9, 2024. Results 1465

#	Searches	Results
#1	"Knee"[Mesh]	164422
#2	knee*[Title/Abstract]	191519
#3	#1 OR #2	195006
#4	arthroplasty[Title/Abstract] OR Joint Prosthesis[Title/Abstract] OR arthroplas*[Title/Abstract] OR surg*[Title/Abstract] OR replac*[Title/Abstract] OR arthrosco*[Title/Abstract] OR operat*[Title/Abstract] OR prosthe*[Title/Abstract] "arthroplasty"[Title/Abstract] OR "joint prosthesis"[Title/Abstract] OR "arthroplas*"[Title/Abstract] OR "surg*"[Title/Abstract] OR "replac*"[Title/Abstract] OR "arthrosco*"[Title/Abstract] OR "operat*"[Title/Abstract] OR "prosthe*"[Title/Abstract]	3885755
#5	#3 和 #4	97708
#6	Total Knee Arthroplasty[Title/Abstract] OR Total Knee Replacement[Title/Abstract] OR Unicompartmental Knee Arthroplasty[Title/Abstract] OR Unicondylar Knee Arthroplasty[Title/Abstract] OR Partial Knee Arthroplasty[Title/Abstract] OR Unicondylar Knee Replacement Partial Knee Replacement[Title/Abstract] OR Unicompartmental Knee Replacement[Title/Abstract]	33860
#7	#5 or #6	97708
#8	"Prostheses and Implants"[Mesh]	5995

		08
#9	Prosthe*[Title/Abstract] OR endoprosthe*[Title/Abstract] OR implant*[Title/Abstract]	601407
#10	#8 or #9	941028
#11	#7 and #10	29859
#12	(((("Artificial Intelligence"[Mesh]) OR "Deep Learning"[Mesh]) OR "Machine Learning"[Mesh]) OR "Neural Networks, Computer"[Mesh]) OR "Algorithms"[Mesh] "Artificial Intelligence"[MeSH Terms] OR "Deep Learning"[MeSH Terms] OR "Machine Learning"[MeSH Terms] OR "neural networks, computer"[MeSH Terms] OR "Algorithms"[MeSH Terms]	453921
#13	algorit*[Title/Abstract] OR artificial Intelligence[Title/Abstract] OR AI[Title/Abstract] OR computational intelligence[Title/Abstract] OR machine intelligence[Title/Abstract] OR computer reasoning[Title/Abstract] OR computer vision*[Title/Abstract] OR machine learnin*[Title/Abstract] OR deep learnin*[Title/Abstract] OR neural network*[Title/Abstract] OR computer-assisted[Title/Abstract] OR computer-aided[Title/Abstract] OR support vector machine*[Title/Abstract] OR support vector network*[Title/Abstract] OR computer methodologies[Title/Abstract] OR automated pattern recognition[Title/Abstract] OR pattern recognition systems[Title/Abstract] OR K nearest neighbour [Title/Abstract] OR kNN[Title/Abstract] OR random forest[Title/Abstract] OR Fuzzy logic[Title/ Abstract] OR Decision tree[Title/Abstract] OR automation[Title/Abstract] OR kernel[Title/Abstract] OR information processing[Title/Abstract] OR predict* model[Title/Abstract] OR reinforcement learning[Title/Abstract] OR supervised learning[Title/Abstract] OR unsupervised learning[Title/Abstract]	741190
#14	#12 or #13	940987
#15	#14 and #11	1465

Database: Cochrane Library

Retrieval date: March 9, 2024. Results 464

#	Searches	Results
#1	MeSH descriptor: [Arthroplasty, Replacement, Knee] explode all trees	3953
#2	MeSH descriptor: [Knee Prosthesis] explode all trees	1002
#3	(Total Knee Arthroplasty or Total Knee Replacement or Unicompartmental Knee Arthroplasty or Unicondylar Knee Arthroplasty or Partial Knee Arthroplasty or Unicondylar Knee Replacement Partial Knee Replacement or Unicompartmental Knee Replacement):ti,ab,kw	9867
#4	#1 or #2 or #3	10298
#5	(knee*):ti,ab,kw	41274
#6	(arthroplasty or Joint Prosthesis or arthroplas* or surg* or replac* or arthrosco* or operat* or prosthe*):ti,ab,kw	410969
#7	#5 and #6	19800
#8	#4 or #7	19800
#9	MeSH descriptor: [Prostheses and Implants] explode all trees	25782
#10	(Prosthe* or endoprosthe* or implant*):ti,ab,kw	255609
#11	#9 or #10	64572
#12	#8 and #11	3710
#13	MeSH descriptor: [Artificial Intelligence] explode all trees	3144
#14	MeSH descriptor: [Deep Learning] explode all trees	311
#15	MeSH descriptor: [Machine Learning] explode all trees	966

#16	MeSH descriptor: [Neural Networks, Computer] explode all trees	616
#17	MeSH descriptor: [Algorithms] explode all trees	7946
#18	(algorit* or artificial Intelligence or AI or computational intelligence or machine int elligence or computer reasoning or computer vision* or machine learnin* or deep le arnin* or neural network* or computer-assisted or computer-aided or support vector machine* or support vector network* or computer methodologies or automated pa ttern recognition or pattern recognition systems or K nearest neighbour or kNN or r andom forest or Fuzzy logic or Decision tree or automation or kernel or informatio n processing or predict* model or reinforcement learning or supervised learning or unsupervised learning):ti,ab,kw	149213
#19	#13 or #14 or #15 or #16 or #17 or #18	150329
#20	#12 and #19	464

Database: Web of Science

Retrieval date: March 9, 2024. Results 2598

#	Searches	Results
#1	TS=(Total Knee Arthroplasty or Total Knee Replacement or Unicompartmental Knee Arthroplasty or Unicondylar Knee Arthroplasty or Partial Knee Arthroplasty or Uni condylar Knee Replacement Partial Knee Replacement or Unicompartmental Knee R eplacement)	23,949
#2	(TS=(arthroplasty or Joint Prosthesis or arthroplas* or surg* or replac* or arthrosco * or operat* or prosthe*)) AND TS=(knee*)	108,839
#3	#1 OR #2	108,839
#4	TS=(Prosthe* or endoprosthe* or implant*)	730,005
#5	#3 AND #4	27,047
#6	TS=(algorit* or artificial Intelligence or AI or computational intelligence or machine intelligence or computer reasoning or computer vision* or machine learnin* or dee p learnin* or neural network* or computer-assisted or computer-aided or support ve ctor machine* or support vector network* or computer methodologies or automated pattern recognition or pattern recognition systems or K nearest neighbour or kNN or random forest or Fuzzy logic or Decision tree or automation or kernel or inform ation processing or predict* model or reinforcement learning or supervised learning or unsupervised learning)	6,585,583
#7	#6 AND #5	2598

Database: EMBASE

Retrieval date: March 9, 2024. Results 1915

#	**Searches**	**Results**
#1	'knee prosthesis'/exp OR 'knee replacement'/exp OR 'knee arthroplasty' /exp	66,311
#2	'knee'/exp	107,244
#3	'knee*':ab,ti	255,912
#4	#2 OR #3	274,434
#5	'arthroplasty':ab,ti OR 'joint prosthesis':ab,ti OR 'arthroplas*':ab,ti OR 's urg*':ab,ti OR 'replac*':ab,ti OR 'arthrosco*':ab,ti OR 'operat*':ab,ti OR ' prosthe*':ab,ti	5,325,933
#6	#4 AND #5	130,789
#7	total knee arthroplasty':ab,ti OR 'total knee replacement':ab,ti OR 'unic ompartmental knee arthroplasty':ab,ti OR 'unicondylar knee arthroplasty ':ab,ti OR 'partial knee arthroplasty':ab,ti OR 'unicondylar knee replace	38,348

	ment':ab,ti OR 'partial knee replacement':ab,ti OR 'unicompartmental knee replacement':ab,ti	
#8	#1 OR #6 OR #7	142,668
#9	'implant'/exp	810,687
#10	'prosthe*':ab,ti OR 'endoprosthe*':ab,ti OR 'implant*':ab,ti	821,508
#11	#9 OR #10	1,311,284
#12	#8 AND #11	46,349
#13	'artificial intelligence'/exp OR 'machine learning'/exp OR 'deep learning'/exp OR 'artificial neural network'/exp OR 'algorithm'/exp	904,717
#14	'algorit*':ab,ti OR 'artificial intelligence':ab,ti OR 'ai':ab,ti OR 'computational intelligence':ab,ti OR 'machine intelligence':ab,ti OR 'computer reasoning':ab,ti OR 'computer vision*':ab,ti OR 'machine learnin*':ab,ti OR 'deep learnin*':ab,ti OR 'neural network*':ab,ti OR 'computer-assisted':ab,ti OR 'computer-aided':ab,ti OR 'support vector machine*':ab,ti OR 'support vector network*':ab,ti OR 'computer methodologies':ab,ti OR 'automated pattern recognition':ab,ti OR 'pattern recognition systems':ab,ti OR 'k nearest neighbour':ab,ti OR 'knn':ab,ti OR 'random forest':ab,ti OR 'fuzzy logic':ab,ti OR 'decision tree':ab,ti OR 'automation':ab,ti OR 'kernel':ab,ti OR 'information processing':ab,ti OR 'predict*:ab,ti model':ab,ti OR 'reinforcement learning':ab,ti OR 'supervised learning':ab,ti OR 'unsupervised learning':ab,ti	895,209
#15	#13 OR #14	1,282,430
#16	#12 AND #15	1915
